# Fungal DNA profiles across maternal and neonatal biological sites: findings from an exploratory pilot study

**DOI:** 10.3389/fmicb.2026.1869806

**Published:** 2026-09-14

**Authors:** Dobrochna Wojciechowska, Sylwia Salamon, Piotr Banachewicz, Sebastian Przemieniecki, Olga Kosewska, Lidia Błaszczyk, Tomasz Szczapa, Katarzyna Wróblewska-Seniuk

**Affiliations:** 1 II Department of Neonatology, Poznan University of Medical Sciences, Poznan, Poland; 2 Doctoral School, Poznan University of Medical Sciences, Poznan, Poland; 3 Department of Plant Microbiomics, Institute of Plant Genetics, Polish Academy of Sciences, Poznan, Poland; 4 Department of Entomology and Phytopathology, Faculty of Environmental Management and Agriculture, University of Warmia and Mazury, Olsztyn, Poland

**Keywords:** early-life microbiome, fungal DNA profiles, *Malassezia*, maternal–infant microbiome, neonatal mycobiome, Oxford nanopore sequencing

## Abstract

**Background:**

The neonatal mycobiome remains an underexplored component of early-life microbial ecology. Although fungi may contribute to immune maturation and host–microbe interactions, fungal DNA profiles across maternal and neonatal body sites are poorly characterized. This exploratory pilot study aimed to characterize fungal DNA profiles across multiple maternal and neonatal biological compartments during early life.

**Materials and methods:**

Ten mother–infant dyads were enrolled at ≥37 weeks’ gestation. Three focal dyads (one complete and two near complete) were selected for detailed mycobiome sequencing, while additional breast milk samples expanded the dataset to 30 analyzed specimens. Maternal (vaginal, rectal, breast milk) and neonatal (skin, oral cavity, stool) samples were collected at 48–72 h after delivery and 6 weeks postpartum. Fungal DNA was analyzed using Oxford Nanopore long-read 18S rRNA sequencing. Taxonomic classification was performed with Kraken2 using the NCBI RefSeq Targeted Loci fungal 18S rRNA database. Alpha- and beta-diversity analyses were conducted in R using the *phyloseq* package.

**Results:**

A total of 428,555 fungal reads representing 107 genus-level operational taxonomic features were retained. Fungal DNA profiles differed across biological sites, with a greater relative abundance of *Malassezia* in neonatal skin and more heterogeneous profiles in gastrointestinal-associated samples. Alpha diversity did not differ significantly between sampling sites, whereas beta diversity suggested exploratory site-associated differences in community composition. Richness-based metrics were higher at 6 weeks than during early sampling, although this trend was not robust after accounting for sequencing depth, and beta-diversity analyses showed no clear temporal shift. Because negative controls were not sequenced, contamination from laboratory or reagent-derived fungal DNA cannot be excluded, and the findings should not be interpreted as evidence of viable fungi or stable colonization.

**Conclusion:**

This exploratory pilot study suggests that fungal DNA profiles differ across maternal and neonatal body sites and that detectable fungal richness may increase by 6 weeks postpartum, although this finding was not robust to sequencing depth. Larger longitudinal studies incorporating ITS-based sequencing, strain-level analyses, sequenced technical controls, and functional profiling are needed to validate these observations and clarify the biological significance of early-life fungal communities.

## 1 Background

The twenty-first century has witnessed a surge of interest in microbiome research across animals and humans. Numerous studies have demonstrated that bacteria and fungi are essential components of the host ecosystem, exhibiting not only pathogenic properties but also beneficial roles in human and animal physiology (Shan et al., 2019). Increasing attention has been directed toward understanding the microbiome of newborns, in whom both environmental and genetic factors shape early microbial colonization.

Maternal microbial transmission before and around birth is influenced by the maternal endometrial, vaginal, gastrointestinal, and oral microbiota and may affect fetal development. The maternal microbiome is shaped by genetic, environmental, dietary and clinical factors, including medication use, infections, and stress. Early microbial colonization is further influenced by mode of delivery, gestational age, feeding practices, and postnatal environmental exposures. Vaginally delivered newborns acquire bacteria specific to the maternal vaginal microbiome, such as *Lactobacillus*, *Sneathia* spp., and *Prevotella*. In contrast, infants born by cesarean section are predominantly colonized with maternal skin-associated taxa, including *Staphylococcus*, *Propionibacterium*, and *Corynebacterium* (Yao et al., 2021).

Preterm birth disrupts normal fetal development, is associated with higher rates of cesarean deliveries, and often leads to intensive medical interventions, factors that collectively predispose to microbial dysbiosis (Arboleya et al., 2022). Human milk contains a distinct microbiota characterized by low bacterial load and high taxonomic diversity, with *Staphylococcus* and *Streptococcus* among the most predominant taxa (Boudry et al., 2021). Recent studies also indicate that skin-to-skin contact modulates microbiota composition and development in early infancy, as evidenced by changes in microbiota volatility and maturation (Eckermann et al., 2024).

Neonates provide unique ecological niches for microbial colonization because their skin, gastrointestinal tract, and oral cavity differ structurally, biochemically, and immunologically from those of adults. Neonatal skin is characterized by an immature epidermal barrier, higher hydration, and dynamic postnatal changes in pH, all of which influence microbial adhesion and colonization patterns (Choi, 2025). Similarly, the neonatal gastrointestinal tract exhibits immature mucosal and immune functions, while the oral cavity is characterized by developing salivary secretion and evolving local immune responses. Consequently, neonatal microbial communities should not be interpreted using adult reference patterns. Instead, microbial colonization during infancy represents a dynamic developmental process shaped by host maturation and environmental exposures.

In addition to bacteria, the human microbiome includes a significant fungal community. However, the mycobiome remains poorly described and under-investigated (Ward et al., 2017). Fungi are estimated to constitute approximately 13% of the adult gut microbiome and encompass more than 100 genera (Schei et al., 2017). Growing evidence suggests that fungal communities contribute to host metabolism, nutrient and vitamin absorption, micronutrient biosynthesis, immune regulation, and maintenance of microbial homeostasis (James et al., 2020; Paul et al., 2020; Mims et al., 2021). Changes in the mycobiome are observed in many medical conditions, including inflammatory bowel disease, colorectal adenomas, pouchitis, and diarrhea, as well as in rare conditions such as graft-versus-host disease and Rett syndrome. However, it remains unclear whether the fungal dysbiosis is a consequence of the disease process or contributes to disease etiology (Huseyin et al., 2017). Therefore, investigating how the human mycobiome evolves from birth to death is extremely important.

Recent advances have refined the concepts of eubiosis and dysbiosis. Rather than being defined by the taxonomic composition of microbial communities, these states are increasingly understood in terms of microbial function, stability, resilience, and interactions with the host. Accordingly, eubiosis reflects a dynamic equilibrium between microorganisms and the host, whereas dysbiosis represents disruption of this balance (Alagiakrishnan et al., 2024; Ma et al., 2024; Safarchi et al., 2025).

Despite growing interest in the mycobiome, significant knowledge gaps remain regarding prenatal and postnatal fungal transmission (Schei et al., 2017). Existing evidence suggests that delivery mode, diet and gestational age at birth influence the infant mycobiome (Ward et al., 2017). However, only a limited number of studies have directly investigated fungal transmission from mother to newborn (Schei et al., 2017; Ward et al., 2018). Although fungal colonization is frequently asymptomatic, opportunistic fungi, particularly *Candida* species, may cause localized or invasive infections in susceptible neonates. Premature infants are especially vulnerable because of immune immaturity, prolonged hospitalization, broad-spectrum antibiotic exposure, and invasive medical procedures. Consequently, fungal infections remain an important cause of morbidity and mortality in neonatal intensive care units (Stewart et al., 2013; Mehler et al., 2022). Several studies also suggest a role for fungi in the development of necrotizing enterocolitis (Stewart et al., 2013).

Furthermore, antibiotic therapy, widely used in neonatal intensive care units, can induce qualitative and quantitative alterations in the mycobiome, promoting the overgrowth of pathobiont species and increasing the risk of fungal infections (Bender et al., 2021). Other interventions affecting mycobiome composition include antifungal drugs and probiotic therapy (Ward et al., 2017). The emergence of multidrug-resistant fungi, such as *Candida auris*, further highlights the clinical importance of understanding fungal colonization and infection in hospitalized neonates (Forsberg et al., 2019). Fungal colonization within the NICU environment is often dominated by a limited number of taxa, likely reflecting routine cleaning and disinfection practices (Boix-Amorós et al., 2019; Heisel et al., 2019). In contrast, breastmilk contains a highly diverse mycobiome whose composition varies according to factors such as geographic location and mode of delivery.

Bacterial–fungal interactions may further influence neonatal microbial colonization and community development. Interactions between *Candida* species and Group B *Streptococcus* have been associated with enhanced microbial persistence and pathogenic potential, highlighting the complexity of neonatal microbial ecosystems (Pidwill et al., 2018).

Advances in molecular biology techniques, including next-generation sequencing (NGS), have greatly improved our ability to elucidate the role of the microbiome, defined as the collective community of all microorganisms - bacteria, fungi, viruses, and archaea—in maintaining health. Emerging machine-learning approaches further underscore the growing diagnostic and biomarker potential of microbiome and mycobiome profiling (Wang et al., 2025; Iyer and Ojcius, 2024). A major milestone in this field was the Human Microbiome Project, conducted by the Human Microbiome Project Consortium. The results, published in Nature in 2012, provided the first comprehensive characterization of the microbiome in healthy adults and established a reference framework for subsequent studies of microbiome variation in health and disease.

To better understand the “normal” neonatal mycobiome and establish baseline data on fungal colonization associated with eubiosis, we conducted a pilot study involving full-term newborns and their mothers. This pilot project was designed to characterize fungal DNA profiles across multiple body sites and to evaluate the feasibility of multi-site mycobiome profiling under clinical conditions.

## 2 Materials and methods

The study was conducted at the Gynecological-Obstetric Hospital of the Poznan University of Medical Sciences between October 2022 and April 2025. It was approved by the local bioethics committee (Resolution No. 834/21 issued by the Bioethics Committee at the Poznan University of Medical Sciences on November 4, 2021). This pilot study was designed to characterize the mycobiome across multiple biological compartments in three focal mother-infant dyads (designated F1, F2, F3), one complete and two near-complete, providing paired maternal and neonatal samples at two time points. Table 1 provides clinical characteristics of the mother–infant dyads included in the sequencing analysis.

**Table 1:** Clinical characteristics of the mother–infant dyads included in the sequencing analysis.

Characteristic	F1	F2	F3
Maternal age (years)	35	27	33
Gestational age (weeks)	39	40	40
Mode of delivery	Vaginal	Vaginal	Cesarean section
Infant sex	Female	Female	Female
Birth weight (g)	3,950	3,180	3,200
Apgar score (1/5 min)	9/10	10/10	10/10
Feeding at 6 weeks	Mixed	Mixed	Exclusive breastfeeding
Complete paired sampling	Near-complete (8/10)	Near-complete (8/10)	Complete (10/10)

In total, 30 specimens were analyzed (Supplementary Table S1). Under the full scheme, each dyad yields ten specimens; F3 provided all ten, whereas F1 and F2 each provided eight (F1 lacked maternal milk at 48–72 h and neonatal stool at 6 weeks; F2 lacked maternal milk at 48–72 h and neonatal stool at 48–72 h), giving 26 dyad specimens. Four additional breast-milk samples extended the milk dataset: two collected at 48–72 h, and two at 6 weeks. The three dyads were chosen for the completeness of paired sampling and adequate sequencing yield, not at random. The exclusion criteria were as follows: maternal colonization with group B *Streptococcus*, antibiotic therapy during pregnancy and in the neonatal period, maternal diabetes mellitus, congenital anomalies, and infant death during the study. Exclusion criteria were selected to minimize factors known to substantially affect maternal or neonatal microbial colonization, including active infections, recent antimicrobial treatment, and clinical conditions requiring intensive medical interventions.

After obtaining written informed consent, we collected the specimens from both mothers and newborns. Initial samples were taken during hospitalization, and follow-up samples were collected during an outpatient visit at six-week visit (6–8 weeks postpartum). Figure 1 illustrates the study design and specifies the samples taken at each time point.

**Figure 1:**
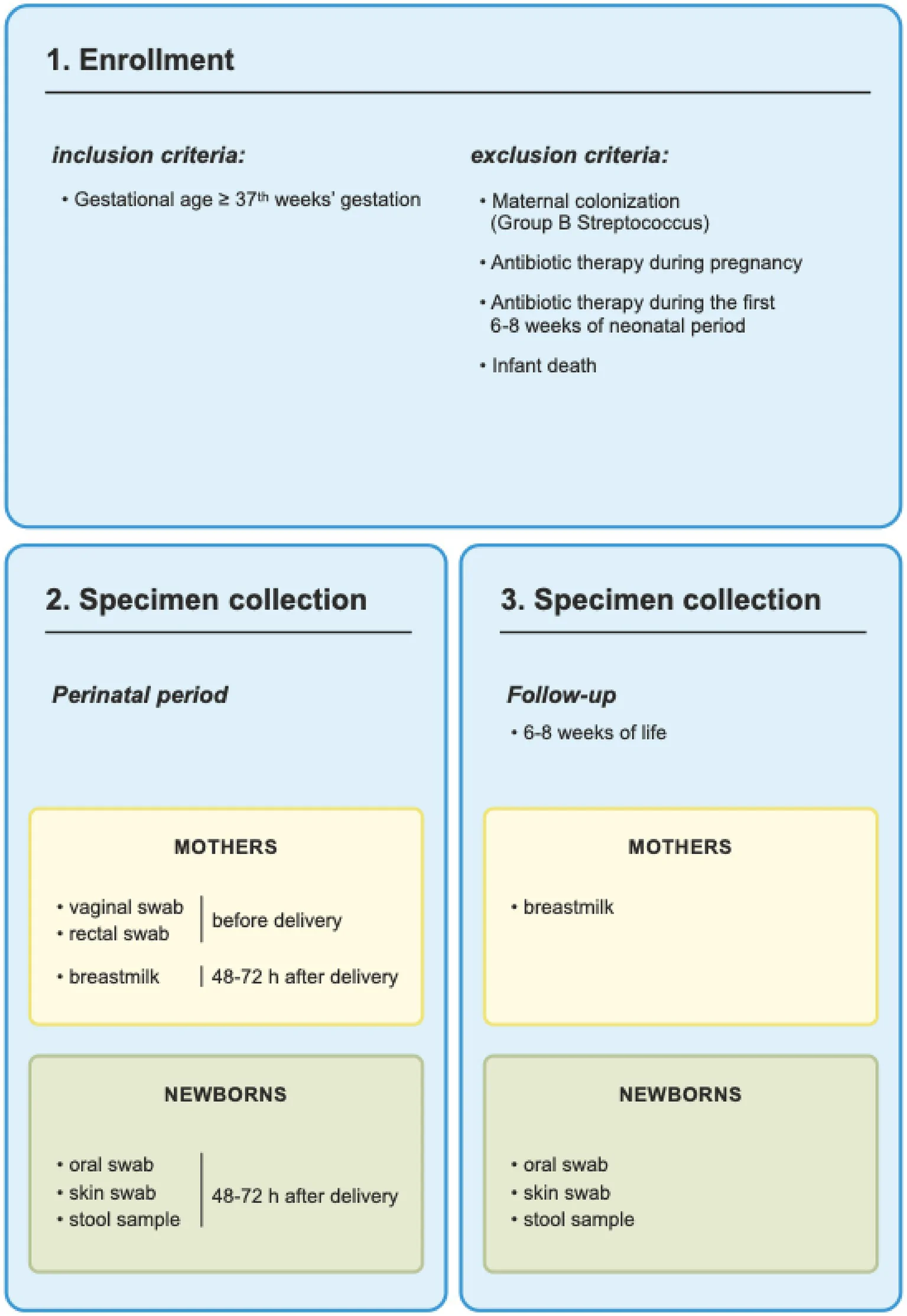
The outline of the conducted study.

The sampling method varied by collection site. Sterile swabs pre-moistened with sterile 0.9% NaCl solution were used for vaginal, rectal, and skin samples. Samples were collected from both mothers and their full-term newborns at two time points: at 48–72 h after delivery and at 6 weeks postpartum. Maternal samples included vaginal swabs, which were obtained from the posterior vaginal fornix shortly before delivery during a sterile speculum examination. Rectal swabs were taken from the anal area shortly before delivery. Breast milk was collected via manual expression under sterile conditions and transferred directly into sterile test tubes. Neonatal samples included skin swabs collected from an approximately 2 cm^2^ area behind the ear. Fecal samples were collected from diapers using a sterile swab and placed into sterile test tubes. In cases where a fecal sample could not be obtained during the clinical visit, caregivers were provided with a sterile at-home collection kit to be returned on ice. All samples were labeled with unique serial numbers and immediately transferred to a laboratory freezer, where they were stored at −80 °C until further analysis.

To minimize the risk of contamination, sample collection and transport were performed using sterile disposable materials protected from external environmental exposure. DNA extraction, PCR setup, and library preparation were carried out using sterile plasticware and dedicated reagents in routinely decontaminated laboratory areas. Work surfaces and laminar flow cabinets were disinfected before and after use and exposed to UV irradiation. DNA extraction, PCR preparation, and library preparation were performed in a class II laminar flow cabinet equipped with ULPA filtration.

Negative controls were included during both the DNA extraction and PCR amplification steps. The absence of visible amplification in the negative controls was verified by agarose gel electrophoresis. These negative controls were not subjected to sequencing, which is acknowledged as a limitation of the study. Therefore, low-abundance, environmental or biologically unexpected taxa were interpreted with caution, and the main descriptions were restricted to dominant genus-level or higher-rank classifier assignments, which are reported as detected fungal DNA rather than as confirmed colonizers.

DNA extraction from swabs and milk samples was performed using the ZymoBIOMICS DNA Mini Prep Kit (Zymo Research, United States) according to the manufacturer’s instructions. One swab or 250 μL of milk was used for each isolation. The 40 min of continuous vortexing on a Multi-Vortex V-32 (Biosan, Poland) during the homogenization step were applied. The QIAamp PowerFecal Pro DNA Kit (Qiagen) was used to isolate DNA from feces. A total of 250 mg of stool was used for each isolation.

The fungal microbiome was analyzed using the primer pair 566-F and 1289-R, which target the 18S small subunit ribosomal RNA (Davidov et al., 2020). Amplification was conducted in two stages. Firstly, PCR was performed using the repliQa HiFi ToughMix 2X (Quantabio, Beverly, Massachusetts, United States) with an annealing temperature of 62 °C. Secondly, the first-stage PCR product was combined with the barcoding primer (PCR Barcoding Expansion 1-12, EXP-PBC001, Oxford Nanopore Technologies) and repliQa HiFi ToughMix 2X according to the conditions used in the first stage. The Ligation Sequencing Kit (SQK-LSK109) (Oxford Nanopore Technologies) and the NEBNext® Companion Module for Oxford Nanopore Technologies® Ligation Sequencing (NEB, Massachusetts, United States) were used to prepare libraries. Libraries were purified using magnetic beads and standardized by fluorometric quantification (Quantus, Promega, Germany). Pooled barcoded samples were sequenced on a Flongle flow cell R10.4.1.

Basecalling was performed on MinKNOW (v2023.06.13) using Guppy v3.2.10 (Wick et al., 2017). The quality of the reads was assessed using FastQC v.0.12.0 (Babraham Bioinformatics, 2023). Adapter sequences were trimmed with Porechop v.0.2.4, with the—*discard_middle* parameter enabled. Basecalled reads were quality- and length-filtered with NanoFilt v2.8.0 (De Coster et al., 2018), retaining reads with a minimum quality score of Q10 and a length between 500 and 1,000 bp (NanoFilt -q 10 -l 500—maxlength 1,000). Filtered reads were classified with Kraken2 v.2.1.64 (Wood et al., 2019) against the NCBI RefSeq Targeted Loci fungal 18S rRNA reference set (fungi.18SrRNA.fna.gz), downloaded and built on 31 October 2025. The 18S rRNA target was selected as a taxonomically broad, well-referenced marker suitable for long-read classification of diverse fungi from low-biomass material. No formal validation of the current sequencing batch or of species-level classification accuracy was performed; any comparisons carried out during method set-up served only as preliminary workflow development. Accordingly, species-level assignments were not relied upon, and all downstream analyses were conducted at the genus level. Classification was run as k2 classify --db 18S_NCBI_targeted --report <sample>.tsv. Per-sample Kraken2 reports were merged with an in-house Python script based on pandas, and a unique identifier was assigned to each genus-level feature from its classification in the Kraken2 report. Because taxonomic confidence decreases as the assigned rank becomes deeper, only features resolved at least to the genus level were retained for downstream analyses; where Kraken2 assigned a species, only the genus-level information was kept. To further reduce the influence of potentially spurious low-abundance sequence features, features were retained only if they reached at least 0.25% relative abundance in at least one sample, following the filtering approach proposed by Reitmeier et al. (2021), using the filter taxa function in the phyloseq package. The 0.25% threshold was originally proposed for 16S bacterial amplicons; as no comparably validated threshold exists for fungal 18S data, it was applied as a pragmatic filter, and its influence on richness estimates was assessed in a sensitivity analysis across four thresholds (unfiltered, 0.1, 0.25, 0.5%; Supplementary Figure S1, Supplementary Data S1), which confirmed that the temporal increase in richness was independent of the threshold.

The structure of the fungal community was analyzed in samples collected from newborns and their mothers. The alpha and beta diversity evaluations, along with the necessary statistical analyses, were conducted using R software version 4.4.2 (2024-10-31) and the phyloseq package (McMurdie and Holmes, 2013). Diversity metrics were computed on unrarefied genus-level counts, because rarefaction discards reads and can remove low-abundance taxa (McMurdie and Holmes, 2014; Willis, 2019). The N3 sample (4 classified reads) was excluded from alpha- and beta-diversity analyses. Alpha diversity was assessed using Observed richness, Chao1 richness estimates, and the Shannon diversity index. Differences in alpha-diversity metrics between time points and sampling sites were evaluated using Wilcoxon rank-sum tests, with Holm correction applied for multiple comparisons (pairwise.wilcox.test); the reported alpha-diversity *p*-values are Holm-adjusted. Because richness estimators are depth-dependent and six-week specimens were sequenced more deeply, the temporal richness comparison was additionally assessed under depth equalization by rarefying all samples to the lowest library size (1,136 reads; 100 independent rarefactions). Robustness to the abundance threshold was assessed across four thresholds (unfiltered, 0.1, 0.25, 0.5%). Chao1 was computed on counts after taxon retention.

Beta diversity was assessed using Bray–Curtis dissimilarity and visualized by principal coordinates analysis. Differences in community composition between groups were evaluated using PERMANOVA with 999 permutations; because specimens were repeated measures from three dyads, permutations for the sampling-site comparison were restricted within dyad (function how() with blocks = dyad, permute package). Homogeneity of group dispersion was assessed using the betadisper function from the vegan package. The additional breast-milk samples from mothers outside the three dyads were included in the site comparison but excluded from the temporal comparison, which was restricted to the paired dyad specimens. Permutations for the temporal test were likewise restricted within dyad. Because maternal follow-up sampling was limited to breast milk, the two time points do not compare the same anatomical compartments; the temporal PERMANOVA is therefore interpreted descriptively rather than as evidence of temporal change. Because the dataset consists of repeated specimens from only three mother–infant dyads, the observations are not independent.

### 2.1 Study-design limitations

This study constitutes an exploratory feasibility pilot, and only three of the ten enrolled dyads were analyzed. The selection of these specimens was not random but was driven by the need to represent both delivery modes and DNA yield, which may have introduced a bias in the dataset toward specimens with higher biomass. Because multiple specimens are analyzed per dyad, the observations are not statistically independent; thus, all tests are exploratory. Richness estimators are depth-dependent, and because six-week specimens were sequenced more deeply, the apparent temporal increase in richness did not persist after rarefaction and is therefore treated as descriptive.

## 3 Results

Sequencing and read processing yielded 767,382 adapter-trimmed reads, of which 604,339 passed quality- and length-filtering, 603,012 were classified by Kraken2, and 439,062 were assigned at least to the genus level. After the 0.25% relative-abundance filter, 428,555 reads were retained for analysis, corresponding to 107 genus-level taxonomic features across 30 specimens. Per-specimen sequencing depth is reported in the sample-inventory table (Supplementary Table S1). Relative abundance profiles showed site-associated differences in fungal community composition across the analyzed sample types (Figure 2, Supplementary Table S2). At the class level, fungal community profiles were dominated by Tremellomycetes and Dothideomycetes across all sampling sites (Figure 2). Tremellomycetes represented the largest relative fraction in milk, oral cavity, and vaginal samples, whereas Dothideomycetes contributed prominently across all sites and represented the largest fraction in anus and stool samples. Skin samples showed a distinct composition compared with the other sites, with a higher relative contribution of Malasseziomycetes. Stool samples also showed a more heterogeneous class-level profile, with larger contributions from Sordariomycetes, Pichiomycetes and the “Other” category. The remaining classes were detected at lower relative abundances across sampling sites.

**Figure 2:**
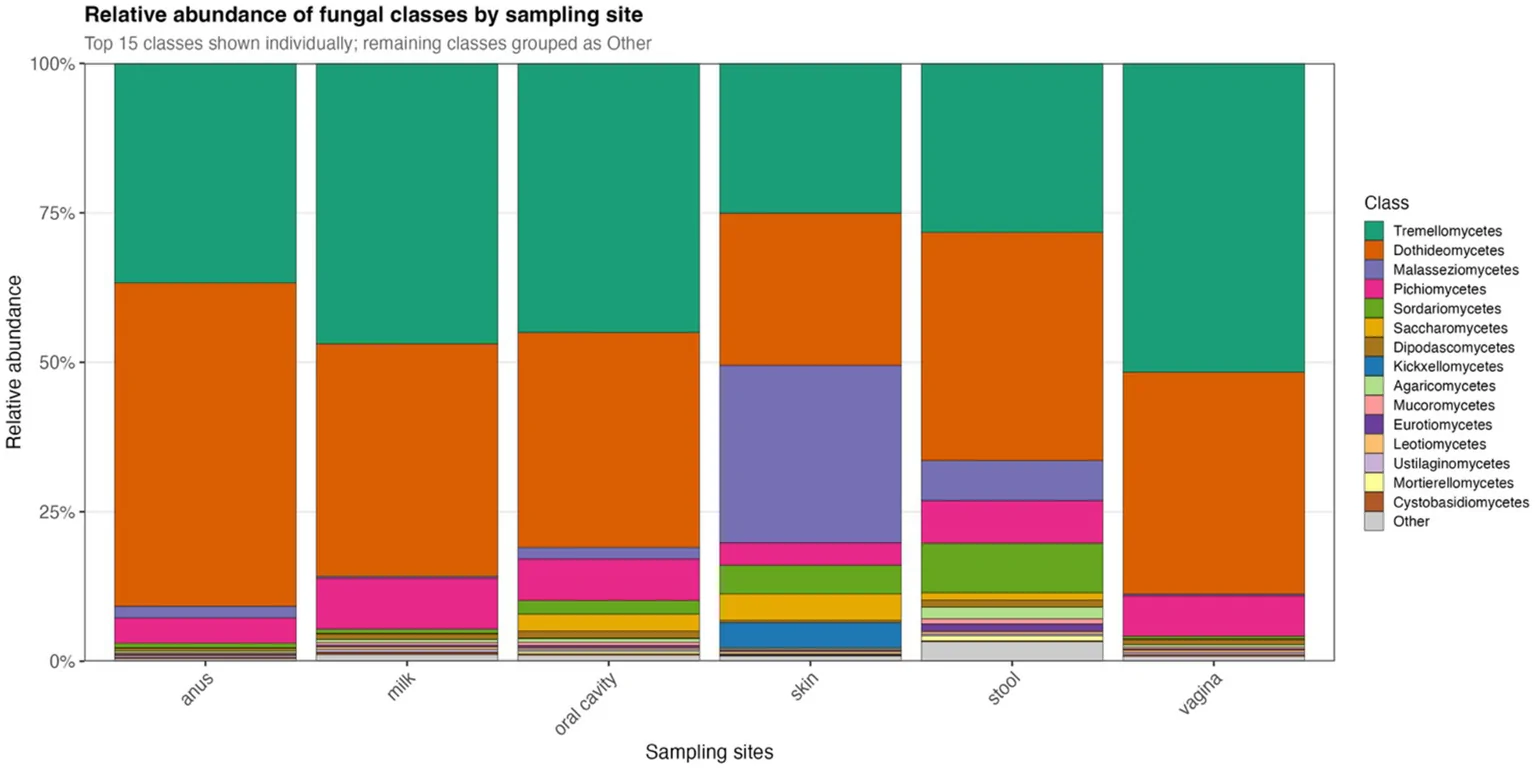
The relative abundance of fungi inhabiting the oral cavity, skin, and stool of newborns, as well as the vagina, anus, and milk of mothers.

Alpha diversity was analyzed based on genus-level features, highlighting observed richness, Chao1 estimates, and the Shannon diversity index (Figure 3). No significant disparities were observed when comparing the samples derived from various biological materials, which suggests that the internal diversity (observed richness and evenness within individual samples) is similar between the groups analyzed. However, beta diversity calculated using Bray–Curtis dissimilarity revealed significant differences among sampling sites (*p* = 0.014, *R*^2^ = 0.28 with permutations restricted within dyads, *p* = 0.036 unrestricted), indicating that genus-level community composition differed between groups. In sensitivity analyses, the site-associated difference was robust to leave-one-sample-out exclusion and to removal of the most isolated observations, but showed the expected loss of power in leave-one-dyad-out testing; it is therefore interpreted as an exploratory, site-associated pattern (Supplementary Table S3, Supplementary Figure S2). These findings suggest that the analyzed biological materials had broadly comparable within-sample diversity but differed in overall community structure.

**Figure 3:**
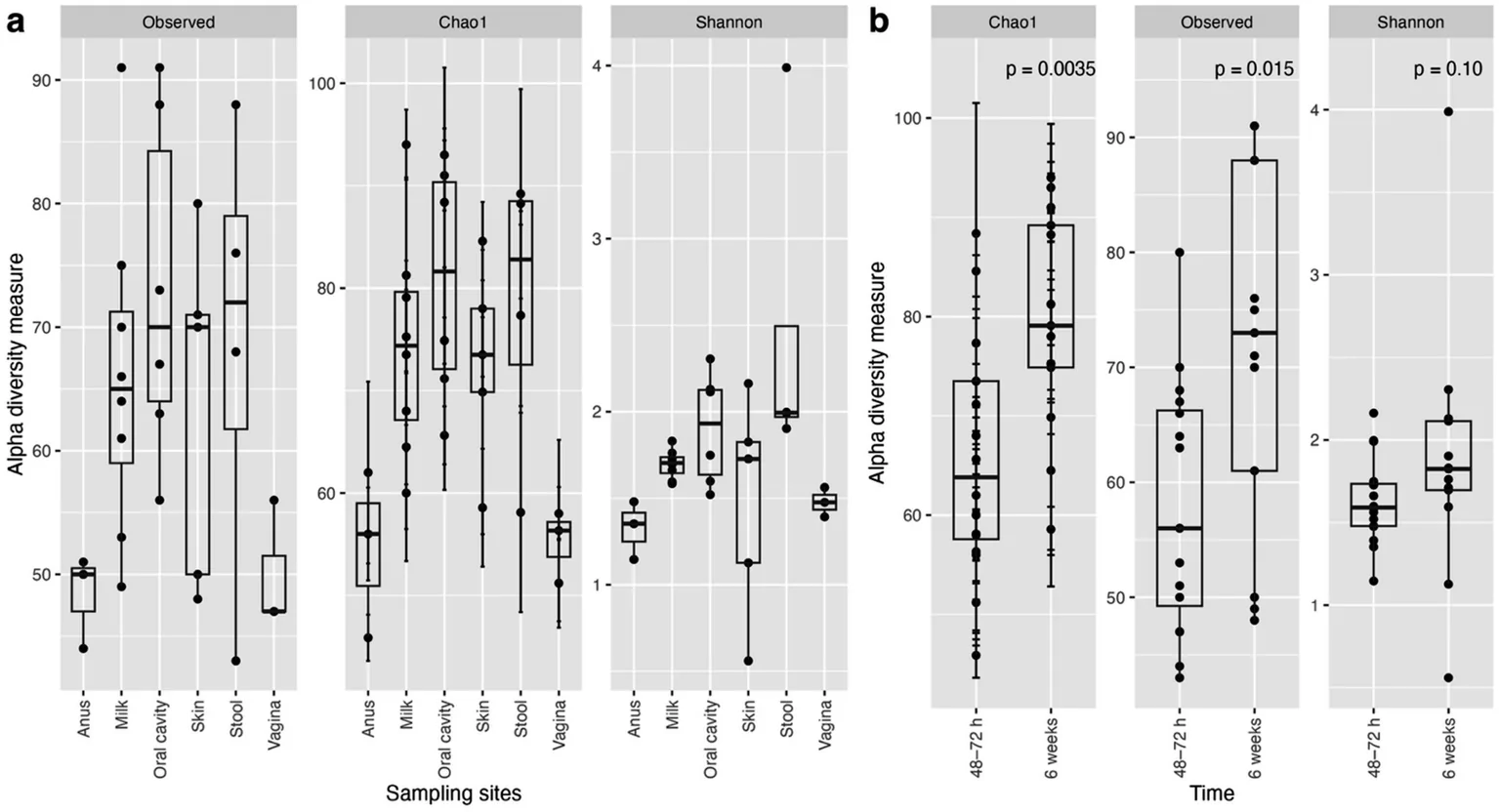
Alpha diversity of fungi colonizing the newborns and their mothers, depending on **(A)** sampling site and **(B)** collection time.

Collection time was associated with differences in richness-based alpha-diversity metrics (Figure 3B). In an unrarefied analysis, both Observed feature count and Chao1 estimates were higher at 6 weeks postpartum than at 48–72 h after delivery (Observed *p* = 0.015; Chao1 *p* = 0.0035; median Observed features: 56 vs. 73; median Chao1: 63.8 vs. 79.1), and this difference was consistent across abundance thresholds (unfiltered, 0.1, 0.25, 0.5%) However, it was not robust to sequencing depth: Observed richness was only weakly correlated with depth (Spearman rho = 0.18, *p* = 0.35), and after rarefaction to the lowest library size the difference was no longer significant (Observed *p* = 0.27, Chao1 *p* = 0.23; mean of 100 rarefactions *p* = 0.11). In contrast, the Shannon diversity index did not differ significantly between time points (*p* = 0.1), suggesting that the apparent temporal difference was driven primarily by the number of detected features rather than by a consistent change in evenness-weighted community diversity. Beta diversity analysis did not show a significant effect of collection time on overall community structure (Figure 4; *R*^2^ = 0.04, *p* = 0.29, permutations restricted within dyad). Because maternal follow-up sampling was limited to breast milk, the two time points do not compare identical anatomical compartments; this temporal contrast should therefore be interpreted with caution. Thus, although richness-based metrics were higher at 6 weeks in the unrarefied data, this difference was not robust to sequencing depth. Homogeneity of dispersion did not differ significantly between sampling sites or time points (betadisper, *p* = 0.335 and *p* = 0.474, respectively), supporting cautious interpretation of the PERMANOVA results.

**Figure 4:**
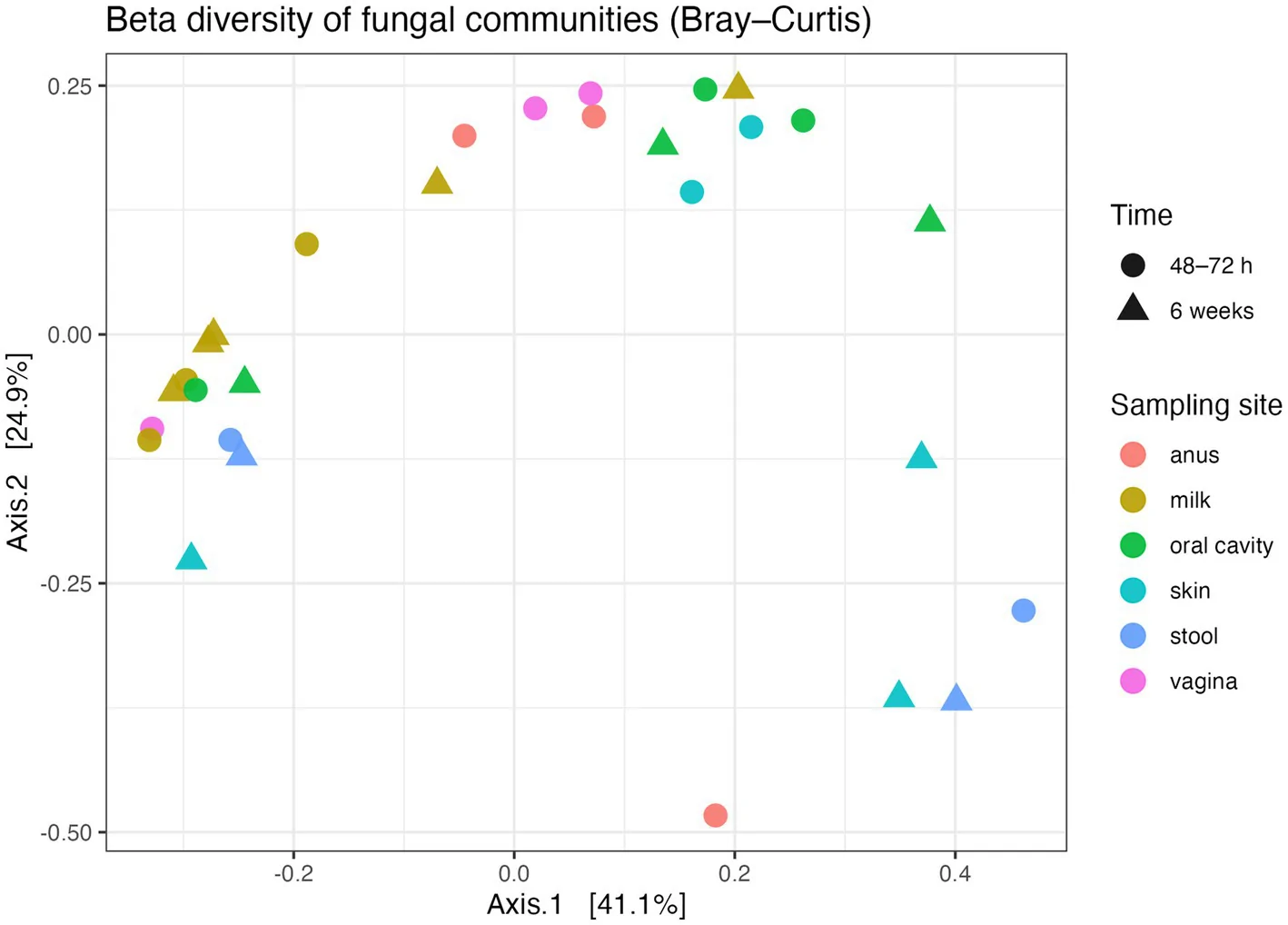
Beta-diversity of fungal communities based on Bray–Curtis dissimilarity, visualized by principal coordinates analysis (PCoA). Each point represents one specimen, colored by sampling site and shaped by collection time (48–72 h after delivery; 6 weeks postpartum).

## 4 Discussion

This pilot study offers an initial assessment of fungal DNA profiles across maternal and neonatal biological sites in the early stages of life. Although alpha-diversity metrics were broadly comparable across sample types, beta-diversity analyses revealed site-associated differences in fungal community composition, indicating distinct fungal DNA profiles across anatomical niches. Class-level analyses showed (Figure 2) that Tremellomycetes and Dothideomycetes predominated across several sample types, suggesting that much of the detected fungal DNA belonged to broad fungal lineages comprising both environmental and host-associated taxa. Given the exploratory design, limited sample size, and methodological constraints of metataxonomic sequencing, these findings represent community-level DNA patterns.

A key finding was the predominance of Malasseziomycetes and the genus *Malassezia* in neonatal skin samples (Figure 2, Supplementary Figure S3), consistent with previous studies identifying *Malassezia* as a characteristic component of the early skin mycobiome (Nagata et al., 2012; Wang et al., 2022). These observations align with reports describing the progressive stabilization of *Malassezia*-dominated skin communities during infancy (Zhu et al., 2020). In our cohort, *Malassezia*-assigned fungal DNA was already prominent at 48–72 h after delivery and remained among the dominant assignments at 6 weeks (Supplementary Figure S4). Because the small sample size precludes statistical inference and the abundance of *Malassezia* was not tested formally within individual dyads, this observation is described only descriptively and is broadly consistent with previous reports of *Malassezia* as a prominent early skin-associated fungal taxon. In the single infant delivered by cesarean section, *Malassezia*-assigned reads were lower (Supplementary Figure S5); as only one infant was delivered this way, this is reported as an individual observation and no delivery-mode effect can be inferred. Delivery mode has nonetheless been reported to shape the early skin and infant mycobiome in adequately powered studies (Wang et al., 2022; Boix-Amorós et al., 2019; Ward et al., 2017) and clarifying whether it influences early skin-associated *Malassezia* DNA will require larger cohorts.

The high contribution of *Malassezia*-assigned taxa in skin samples was consistent with the site-associated patterns observed in beta-diversity analyses, suggesting that neonatal skin carries a distinct fungal DNA profile compared with oral, gastrointestinal, and maternal sites. In contrast, stool samples displayed greater taxonomic heterogeneity, with a larger contribution of low-abundance fungal classes grouped as “Other,” potentially reflecting environmental and dietary exposures, transient fungal DNA, or limited taxonomic resolution in low-biomass samples.

Distinct fungal profiles were also observed across neonatal oral and gastrointestinal-associated samples. (Figure 2, Supplementary Figure S3). However, these findings should not be interpreted as evidence of direct maternal–infant transmission, as they may equally reflect shared environmental, dietary, or perinatal exposures. Similar interpretations were proposed by Heisel et al. (2019), who demonstrated that breast milk and NICU environmental surfaces contribute fungal taxa to the developing infant mycobiome.

Likewise, Olm et al. showed that fungal community assembly during early life is influenced by both maternal and environmental reservoirs (Olm et al., 2018). The sporadic detection of *Saccharomyces*, observed at high abundance in only a single oral sample collected at 6 weeks, further suggests that some fungal taxa may represent transient rather than stable members of early-life fungal communities.

Notably, several of the most abundant classifier-assigned genera, such as Sugitazyma and Superstratomyces, correspond to environmental or plant-related fungi rather than typical human-associated taxa. These assignments may result from database limitations, environmental exposure, transient DNA or laboratory/reagent background; the present design cannot distinguish among these possibilities.

Beyond these dominant assignments, several environmentally associated or biologically unexpected classifications, including *Mangifericomes*, *Brunneoclavispora*, *Ustilaginoidea*, *Pyrenidium*, *Pseudallescheria*, and *Trichoderma*, were also detected. These taxa may reflect environmental or dietary exposure, transient fungal DNA, classification uncertainty, or background contamination. Similar observations have been reported in other low-biomass mycobiome studies, in which environmental fungal DNA contributes substantially to the detected diversity (Eisenhofer et al., 2019). Although alpha-diversity metrics (observed features, Chao1 richness, and Shannon diversity) did not differ significantly between biological sample types, beta-diversity analyses showed site-associated differences in fungal community composition. These findings suggest that fungal DNA profiles were more strongly associated with anatomical location than with overall within-sample diversity. Such patterns may reflect differences in local physiological conditions, including moisture, pH, lipid availability, immune activity, and environmental exposure. Similar niche-specific organization of the human mycobiome has been reported by Belvoncikova et al. (2022), while Nilsson et al. (2019) and Lu et al. (2023) emphasized the importance of improved fungal taxonomic resolution for characterizing fungal diversity in complex biological environments.

Longitudinal analyses suggested a higher number of detected fungal taxa by 6 weeks of life, as reflected by higher observed richness and Chao1 estimates, whereas Shannon diversity remained unchanged. However, because six-week specimens were sequenced more deeply and this difference did not persist after rarefaction to equal library size, we interpret it as a depth-sensitive, descriptive observation rather than a firm developmental trend. Beta-diversity analyses demonstrated only a non-significant temporal trend, indicating that additional fungal taxa became detectable over time while the dominant community structure remained broadly stable. Similar temporal patterns have been described in developing infant gut and skin mycobiomes (Turunen et al., 2023; Zhu et al., 2020). The apparent increase in detected richness without significant community-wide restructuring would be consistent with ongoing fungal community assembly from multiple sources during early life.

The observed site-associated fungal DNA profiles support the concept that early-life fungal communities are shaped by local anatomical and environmental conditions, consistent with previous observations by Ward et al. (2017). While bacterial community development has been extensively investigated, the present findings highlight the need for further studies examining fungal community organization during early life. Rather than identifying specific fungal biomarkers, our findings provide a description of fungal DNA profiles across maternal and neonatal sites. The observed taxonomic overlap between maternal and neonatal samples (Supplementary Figure S6) highlights the complexity of fungal community assembly during early life and the difficulty of distinguishing maternal from other potential sources.

From a methodological perspective, long-read 18S rRNA amplicon sequencing, combined with taxonomic classification using the NCBI RefSeq Targeted Loci Project fungal 18S rRNA reference database, provided a valuable framework for the characterization of fungal DNA profiles. However, the identification of unexpected taxa highlights the limitations of this approach for human mycobiome research. Future investigations would benefit from ITS1- or ITS2-based sequencing using the UNITE database, along with mock communities, sequenced negative controls, and targeted validation of clinically or biologically unexpected taxa.

Several limitations constrain interpretation. Because extraction and PCR negative controls were not sequenced, reagent- and laboratory-derived fungal DNA cannot be identified or excluded, and low-abundance or biologically unexpected taxa should be viewed with particular caution. Profiling relied on long-read 18S rRNA sequencing analyzed at the genus level; species-level assignments were not used or formally validated, and no ITS-based confirmation was performed, which limits taxonomic resolution. All assignments represent detected fungal DNA and cannot be taken as evidence of viable fungi or established colonization. Only one infant was delivered by cesarean section, so delivery mode could not be assessed. Finally, where maternal and neonatal samples shared genus-level assignments, the design cannot establish whether this reflects maternal transfer, shared environmental exposure, laboratory background, or chance.

In summary, this pilot study provides a preliminary characterization of fungal DNA profiles across maternal and neonatal biological sites during early life and demonstrates both the feasibility and the current limitations of multi-site fungal profiling from low-biomass clinical specimens using long-read 18S rRNA sequencing. Within these constraints, the predominance of *Malassezia*-assigned reads in neonatal skin indicates that fungal DNA profiles vary across anatomical niches, and detectable richness appeared to increase over the first weeks of life, although this was not robust to sequencing depth. This pilot contributes on two levels. It provides an internally consistent, literature-concordant descriptive baseline of early-life fungal DNA profiles, most notably *Malassezia* predominance in neonatal skin; and, methodologically, it delineates where multi-site 18S profiling of low-biomass maternal and neonatal specimens is informative and where it is not, offering practical guidance for future human mycobiome studies. On this basis, it establishes a workflow and testable hypotheses for larger longitudinal studies incorporating ITS-based sequencing, strain-level resolution, functional analyses, and sequenced technical controls.

## Data Availability

The datasets presented in this study can be found in online repositories. The names of the repository/repositories and accession number(s) can be found at: https://www.ncbi.nlm.nih.gov/, PRJNA1459286.

## Supplementary material

SUPPLEMENTARY TABLE S1 Sample inventory and per-specimen sequencing depth.

SUPPLEMENTARY TABLE S2 Genus-level taxonomic profiles.

SUPPLEMENTARY TABLE S3 PERMANOVA sensi=vity analyses.

SUPPLEMENTARY DATA S1 Per-specimen richness across filtering thresholds.
